# 

**Published:** 1886-02

**Authors:** 


					﻿hole Number, 314.
•FEBRUARY, I886.
/c^LiL.jAlamter 2
THE
CHICAGO MEDICAL JOURNAL AND EXAMINER
EDITORS:
S. J. JONES, M.D., LL.D.
W. W. JAGGARD, A.M., M.D.
HAROLD N. MOYER, M.D.
PUBLISHED MONTHLY by
OLARK & LOKGLEY,
Under direction of
THE CHICAGO MEDICAL PRESS ASSOCIATION,
163-165 Dearborn Street.
				

